# Supplementary material for: DNA methylation patterns facilitate tracing the origin of neuroendocrine neoplasms
Source: Nat Commun. 2025 Oct 27;16:9477. doi: 10.1038/s41467-025-65227-8 (PMC12559432; doi:10.1038/s41467-025-65227-8)
Supplement: Supplementary file 1 — Supplementary Information [file 41467_2025_65227_MOESM1_ESM.pdf]

Supplementary Information

DNA methylation patterns facilitate tracing the origin of neuroendocrine neoplasms

Benjamin Goeppert *et al.*

Supplementary Figures **2**

Supplementary Figure 1: Hepatic NEN without known primary are associated with poor outcome. 2

Supplementary Figure 2: DNA methylation analysis reveals that gastric, duodenal and colorectal NEN do not exhibited clear clusters. 4

Supplementary Figure 3: Paired primary NEN and NEN metastases exhibit similar epigenetic profiles. 5

Supplementary Figure 4: SNP distance confirmed close distance of the 12 paired samples.. 6

Supplementary Figure 5: DNA methylation analysis reveals that large- and small-cell type NEC clustered together. 7

Supplementary Figure 6: Genomic copy number profiles of NET and NEC. 8

Supplementary Figure 7: Copy number profiles of liver tumors. 9

Supplementary Figure 8: Copy number profiles of primary NEN of different organ sites. 10

Supplementary Figure 9: Proportion of latent methylation components (LMC) in NEN of different organ sites. 11

Supplementary Figure 10: DNA methylation classifier can accurately predict the NEN organ site. 12

Supplementary Figure 11: Comparison of the Random Forest and XGBoost prediction algorithms. 13

Supplementary Figure 12: Copy number profiles of primary NEN based on predicted organ site. 14

Supplementary Figure 13: Proportion of Leukocytes Unmethylation for Purity (LUMP) in NEN of different organ sites. 15

Supplementary Figures

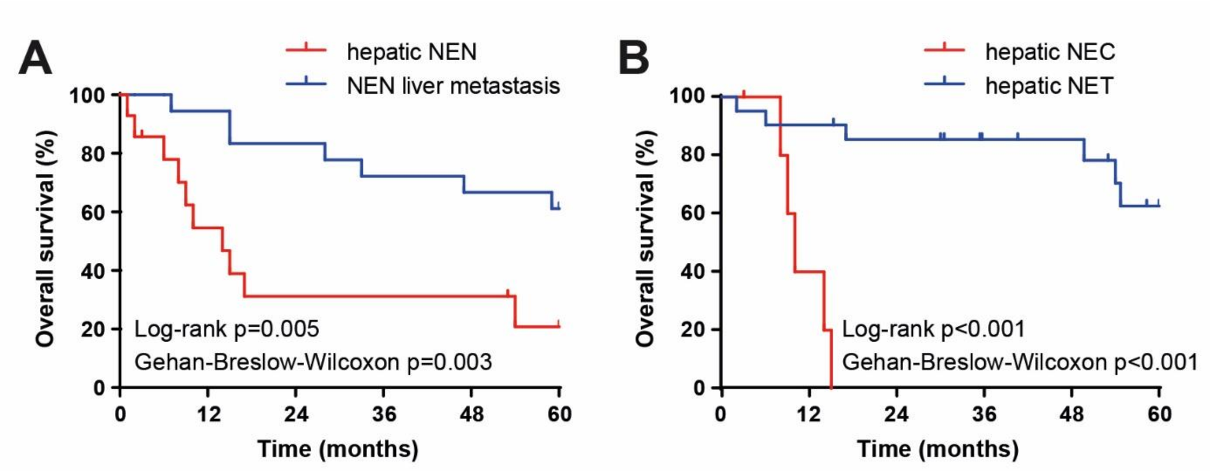

Supplementary Figure 1: Hepatic NEN without known primary are associated with poor outcome.

(A) Kaplan-Meier survival curve of hepatic NEN without known primary tumor (N=14) and of NEN liver metastases of known primary (N=20). **(B)** Kaplan-Meier survival curve of hepatic NEC (N=6) and hepatic NET (N=21) without known primary of the discovery and the validation cohort.

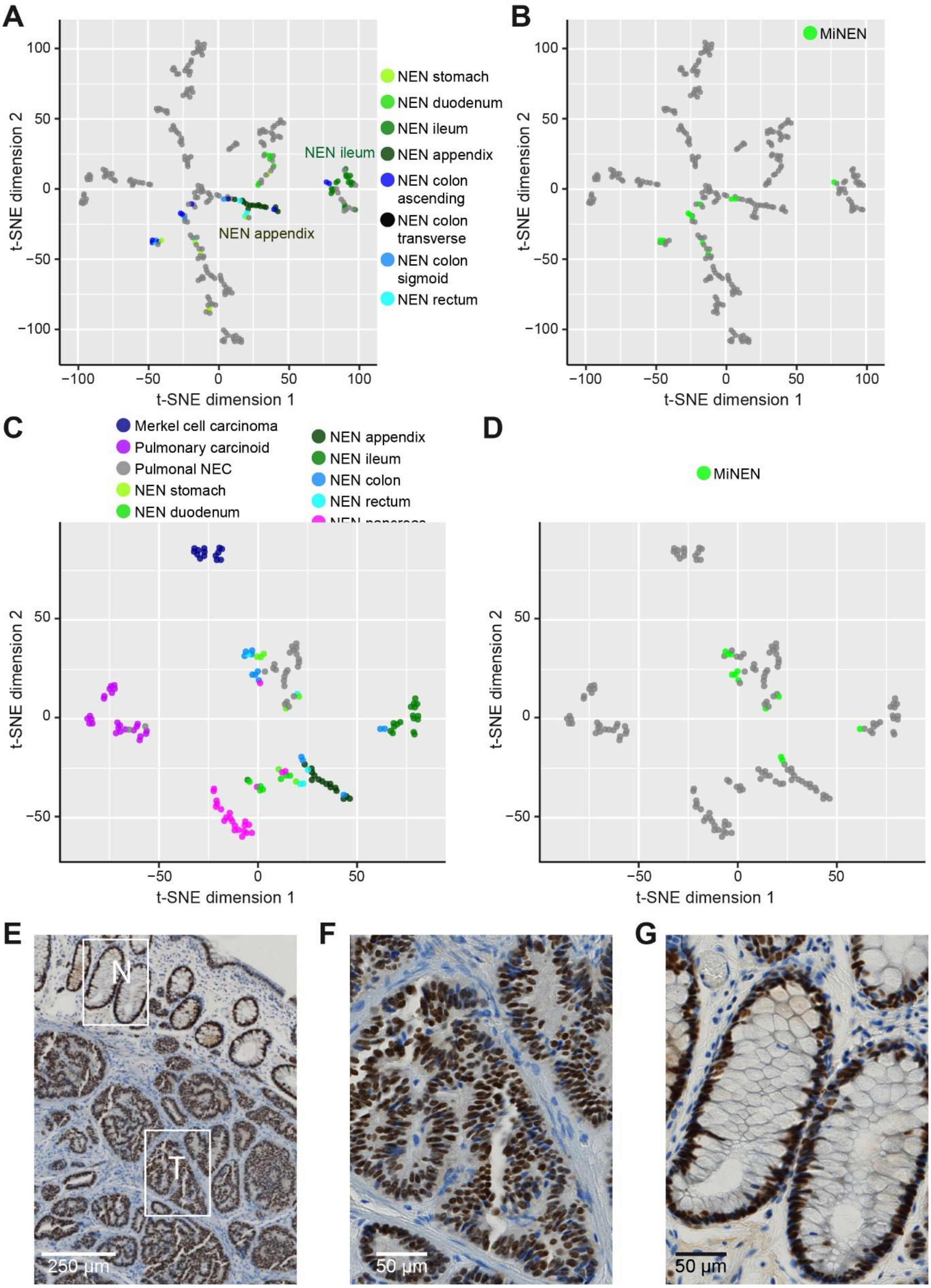
A
B
C
D
E
F
G
t-SNE dimension 1
t-SNE dimension 2
NEN stomach
NEN duodenum
NEN ileum
NEN appendix
NEN colon ascending
NEN colon transverse
NEN colon sigmoid
NEN rectum
MiNEN
Merkel cell carcinoma
Pulmonary carcinoid
Pulmonal NEC
NEN colon
N
T
250 µm
50 µm
50 µm

Supplementary Figure 2: DNA methylation analysis reveals that gastric, duodenal and colorectal NEN do not exhibited clear clusters.

(A) t-SNE plot of the DNA methylation profiles of CCA, HCC and a total of 198 NEN samples from different organ sites. Indicated are NEN of the stomach (N=7), duodenum (N=7), ileum (N=18), appendix (N=15), ascending colon (N=9), transverse colon (N=1), sigmoid colon (N=3) and rectum (N=5). **(B)** Indicated are the 12 MiNEN (2 gastric and 10 colorectal) which were included in this study. Only the neuroendocrine component of the MiNEN was analyzed. **(C)** t-SNE plot of the DNA methylation profiles of the 150 patients with NEN from defined organ sites. Merkel cell carcinomas (N=14 tissues of 11 patients), pulmonary carcinoids (N=25), pulmonary NEC (N=24), gastric NEN (N=7), duodenal NEN (N=7), appendiceal NEN (N=15), ileal NEN (N=18), colon NEN (N=13), rectal NEN (N=5) and pancreatic NEN (N=25) are indicated. **(D)** Furthermore, the 12 MiNEN are indicated in the t-SNE of 150 patients with NEN from defined organ sites. **(E)** Colorectal MiNEN (N=8), MiNEN of the stomach (N=2), ileal NEN (N=10), pancreatic NEN (N=10), pulmonal NEC (N=10) and pulmonary carcinoids (N=10) were analyzed for SATB2 immunoreactivity (Supplementary Data 1). Representative images of SATB2 immunohistochemical staining of a colorectal NET G2 showing positive nuclear staining in the tumor (T) and normal (N) tissue. **(F)** Magnified images of the tumor and **(G)** normal tissue are shown.

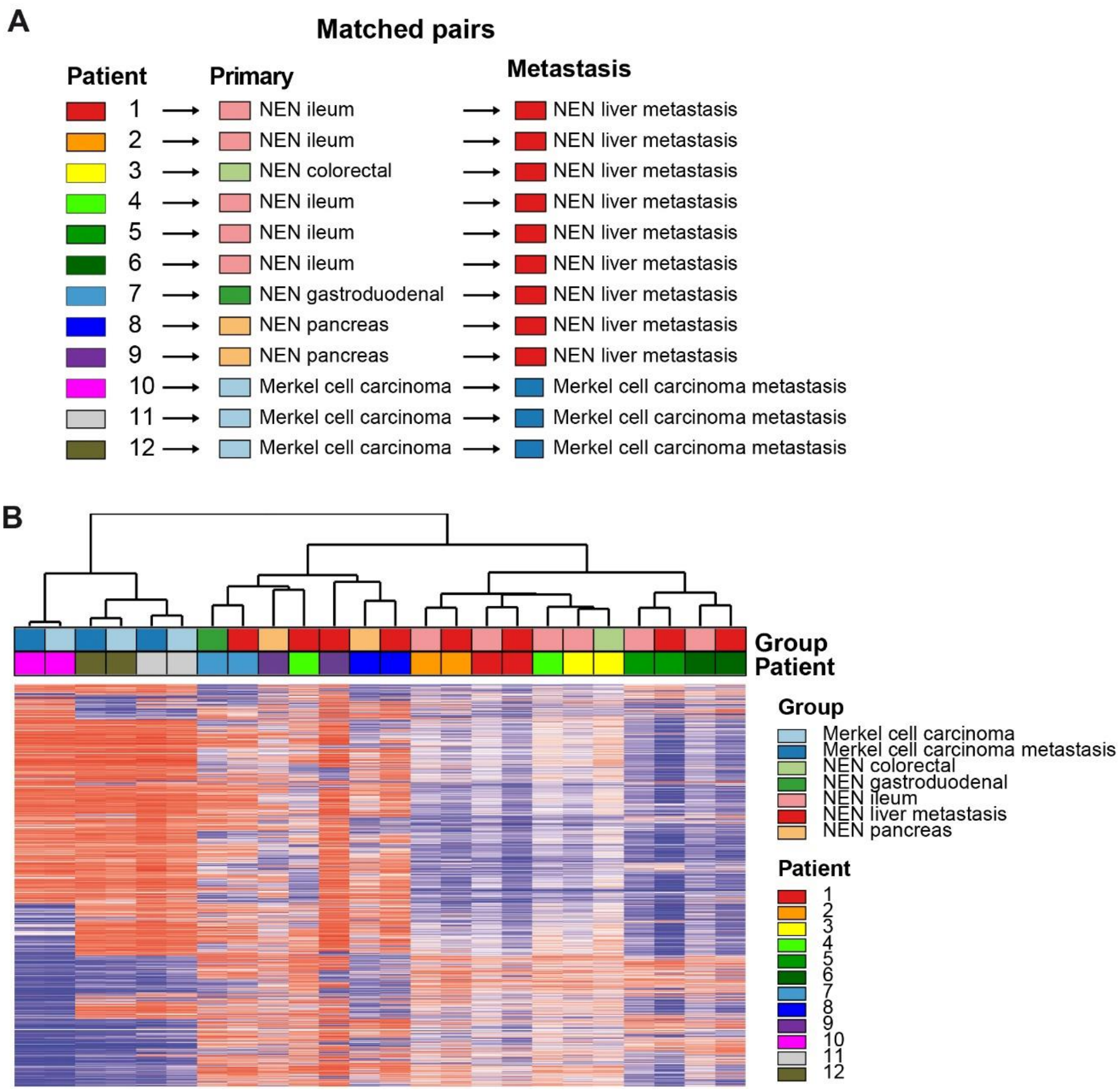

Supplementary Figure 3: Paired primary NEN and NEN metastases exhibit similar epigenetic profiles.

Hierarchical clustering of paired primary NEN and corresponding metastasis applying Euclidean ward squared distances (N=12). **(A)** Detailed color assignment of matched pair samples including patient number, primary tumor and metastasis. **(B)** Heatmap displaying similarities of DNA methylation profiles including hierarchical clustering of samples.

A

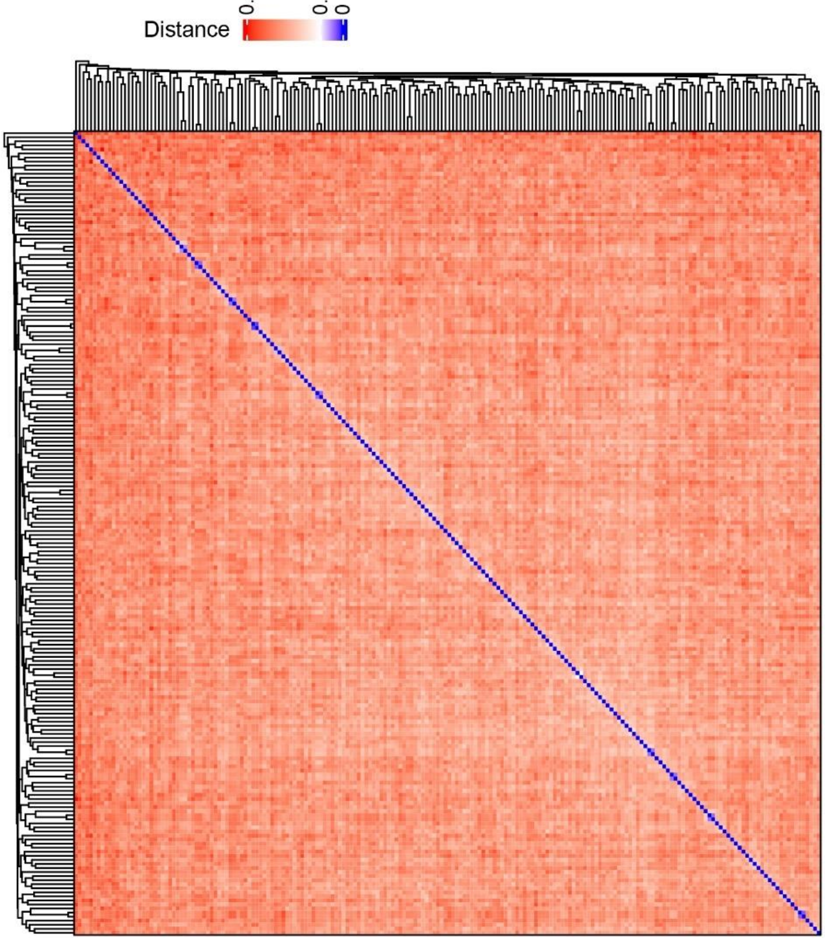

B

Patient	Distance
Patient#001	0.039
Patient#002	0.108
Patient#003	0.032
Patient#004	0.043
Patient#005	0.043
Patient#006	0.076
Patient#007	0.035
Patient#008	0.055
Patient#009	0.079
Patient#010	0.018
Patient#011	0.041
Patient#012	0.043

Supplementary Figure 4: SNP distance confirmed close distance of the 12 paired samples.

(A) Heatmap depicting the pairwise SNP distance of all NEN samples of the Heidelberg cohort. Blue coloring indicates paired samples with a SNP distance below 0.11. **(B)** The distance values between paired samples are shown.

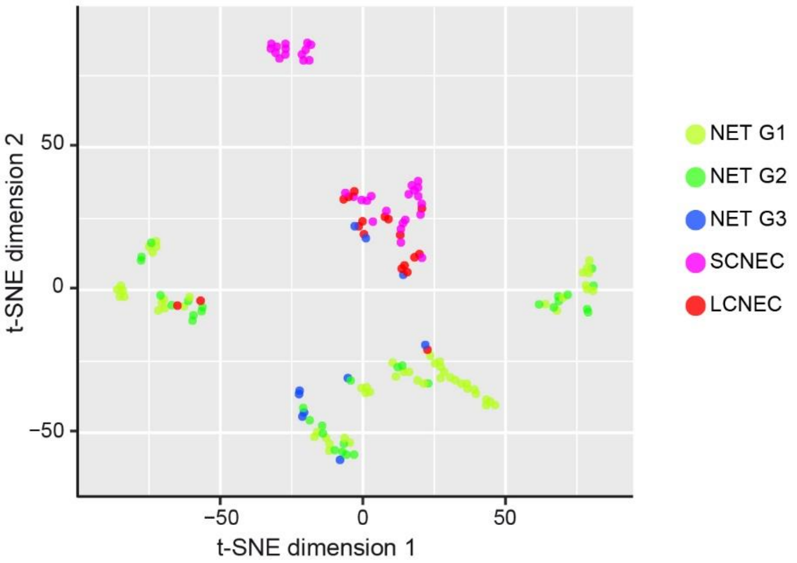

Supplementary Figure 5: DNA methylation analysis reveals that large- and small-cell type NEC clustered together.

Depicted is the t-SNE plot of the DNA methylation profiles of all 150 patients with NEN from defined organ sites. NEN liver metastasis and hepatic NEN were not included for clarity. Merkel cell carcinomas (N=14 tissues of 11 patients), pulmonary carcinoids (N=25), pulmonary NEC (N=24), gastric NEN (N=7), duodenal NEN (N=7), appendiceal NEN (N=15), ileal NEN (N=18), colon NEN (N=13), rectal NEN (N=5) and pancreatic NEN (N=25) are indicated. Across NEN tumor entities, NET G1, NET G2, NET G3, small-cell type NEC (SCNEC) and large-cell type NEC (LCNEC) are shown.

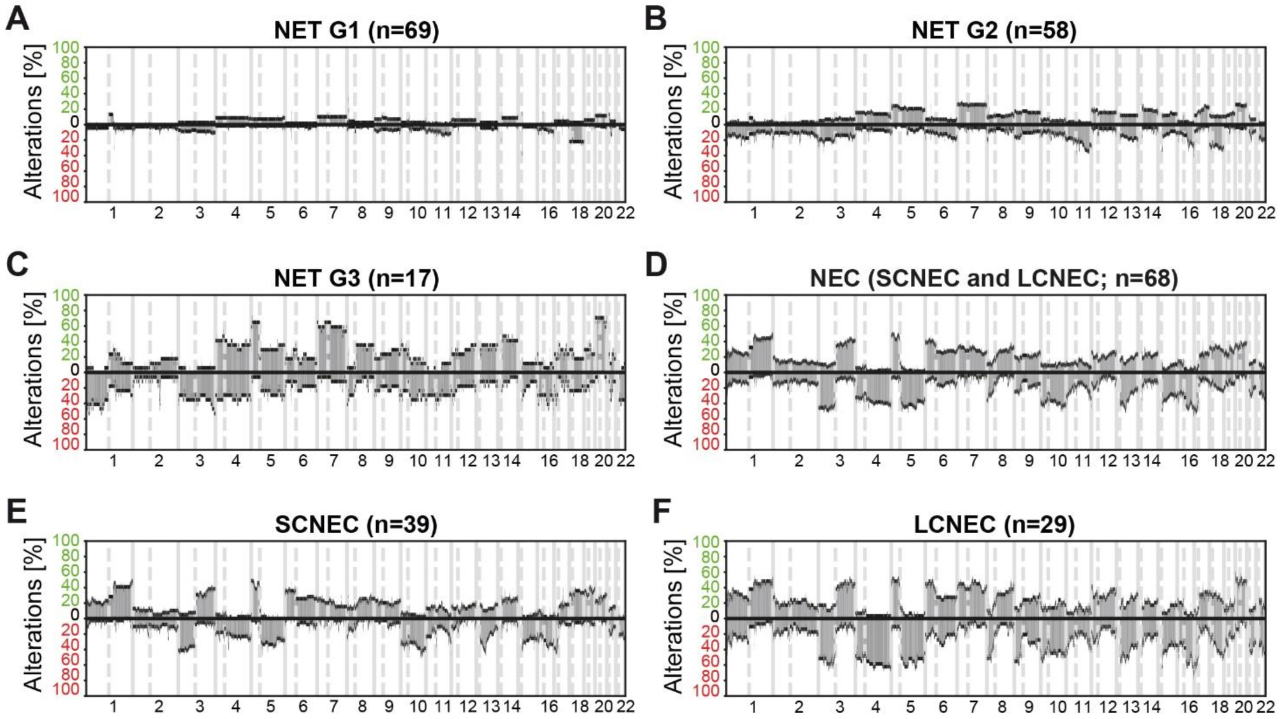

Supplementary Figure 6: Genomic copy number profiles of NET and NEC.

(A) Copy number alterations of NET G1 (N=69), **(B)** NET G2 (N=58), **(C)** NET G3 (N=17), **(D)** all NEC cases (N=68), as well as **(E)** SCNEC (N=39) and **(F)** LCNEC (N=29) separately.

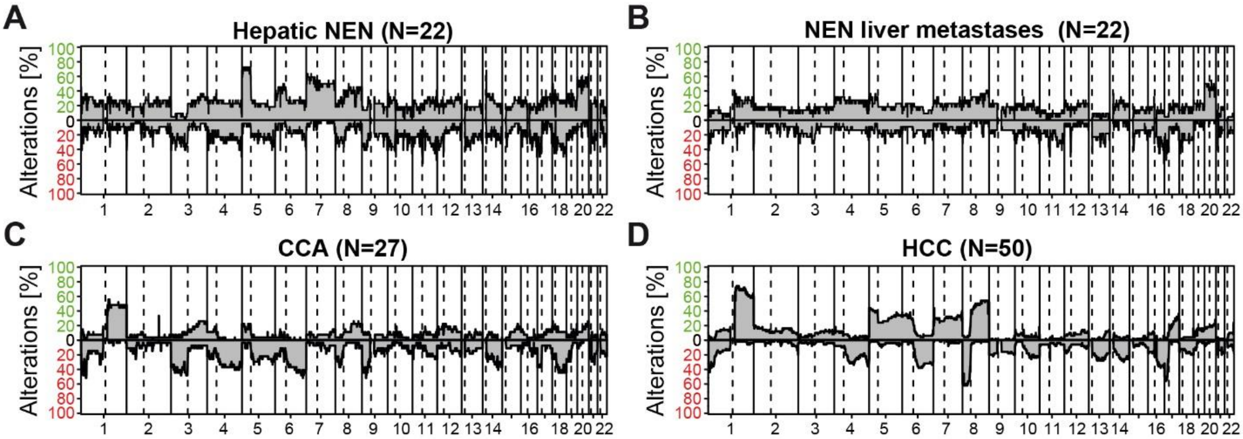

Supplementary Figure 7: Copy number profiles of liver tumors.

(A) Copy number alterations of hepatic NEN (N=22), **(B)** NEN liver metastases (N=22), **(C)** CCA (N=27), **(D)** HCC (N=50).

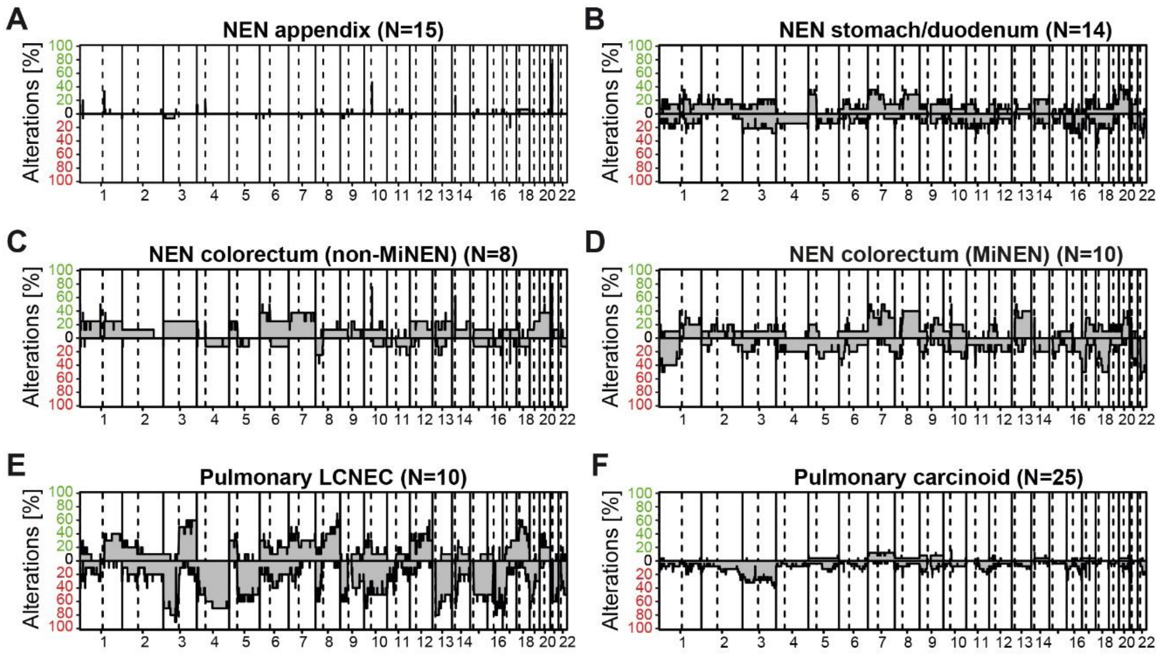

Supplementary Figure 8: Copy number profiles of primary NEN of different organ sites.

(A) Copy number alterations of NEN of the appendix (N=15), **(B)** stomach/duodenum (N=14), **(C)** colorectum excluding MiNEN (non-MiNEN of colorectum; N=8), **(D)** colorectal MiNEN (N=10), **(E)** pulmonary large-cell neuroendocrine carcinoma (LCNEC; N=10) and **(F)** pulmonary carcinoid (N=25). The relative frequency of observed gains (green) and losses (red) are depicted above and below the horizontal line, respectively.

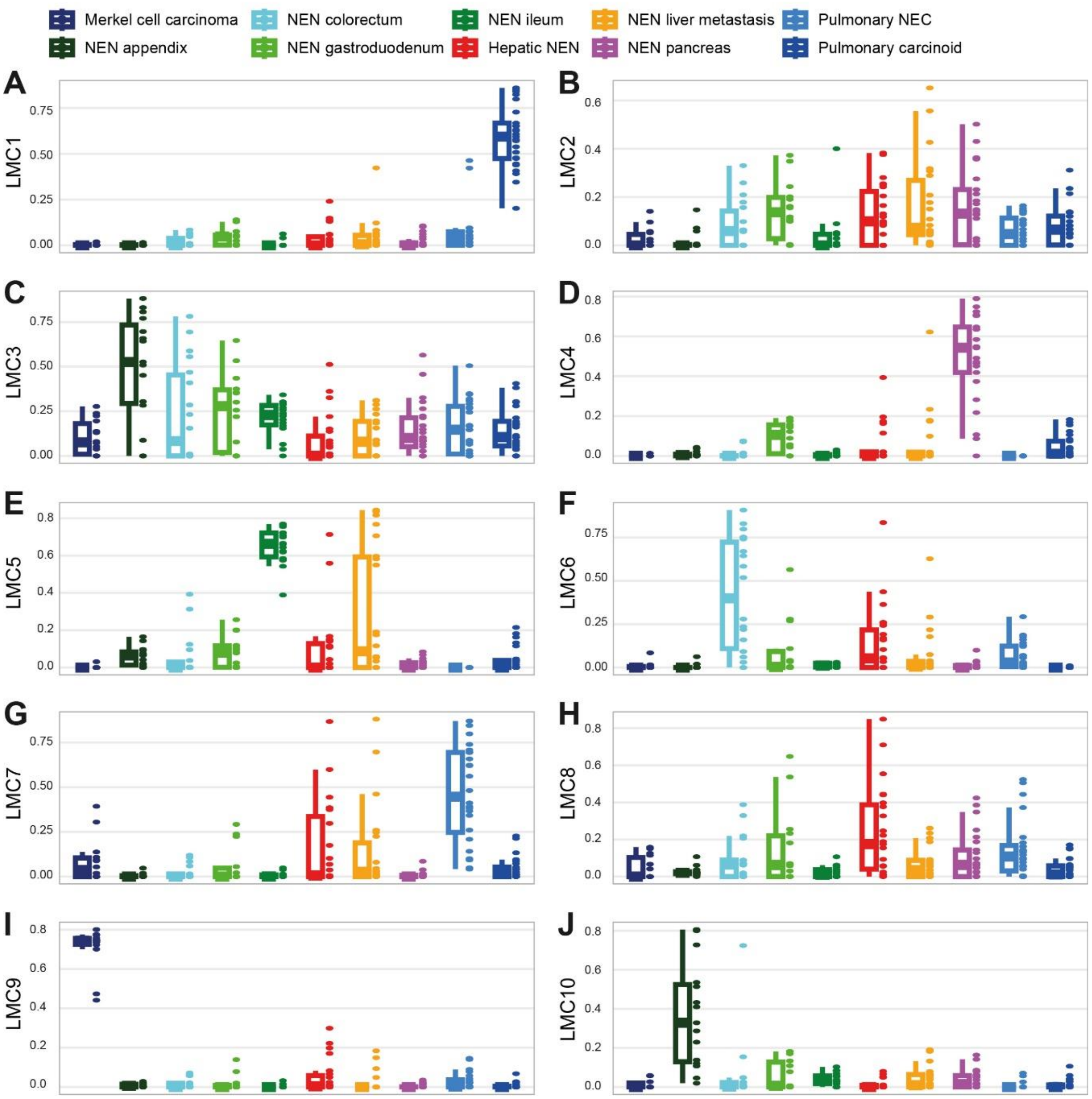

Supplementary Figure 9: Proportion of latent methylation components (LMC) in NEN of different organ sites.

(A-J) Presence of LMC1 to LMC10 in the DNA methylation profiles of Merkel cell carcinoma, colorectal NEN, Merkel cell carcinomas (N=14), appendiceal NEN (N=15), colorectal NEN (N=18), gastric/duodenal (N=14), ileal NEN (N=18), hepatic NEN without known primary tumor (N=27), NEN liver metastases of known primary (N=18), pancreatic NEN (N=25), pulmonary NEC (N=24) and pulmonary carcinoids (N=25), as indicated.

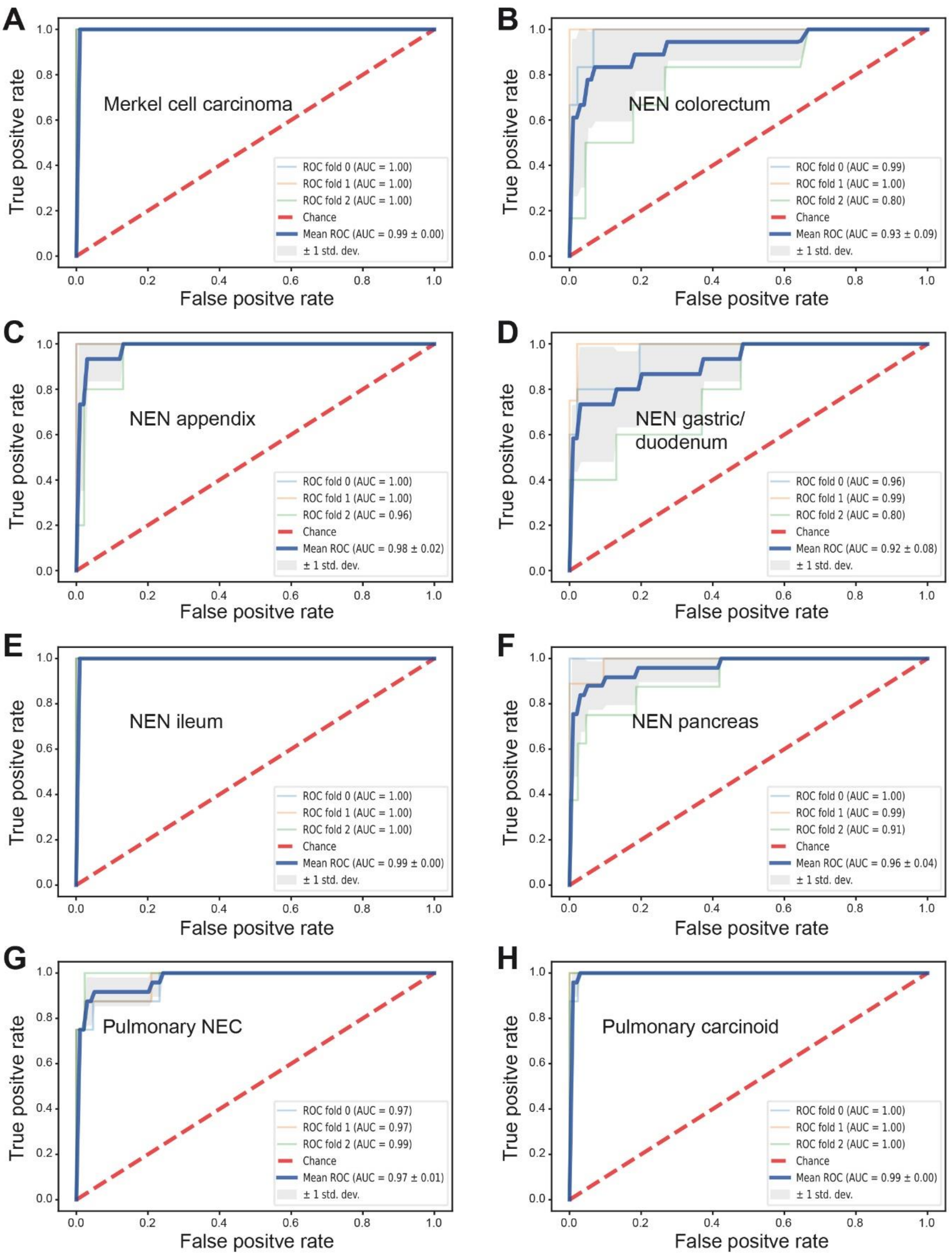

Supplementary Figure 10: DNA methylation classifier can accurately predict the NEN organ site.

(A-H) Receiver operator characteristics (ROC) curves depicting the classifier performance for **(A)** Merkel cell carcinomas (N=14), **(B)** colorectal NEN (N=18), **(C)** appendiceal NEN (N=15), **(D)** gastric/duodenal NEN (N=14), **(E)** ileal NEN (N=18), **(F)** pancreatic NEN (N=25), **(G)** pulmonary NEC (N=24) and **(H)** pulmonary carcinoids (N=25).

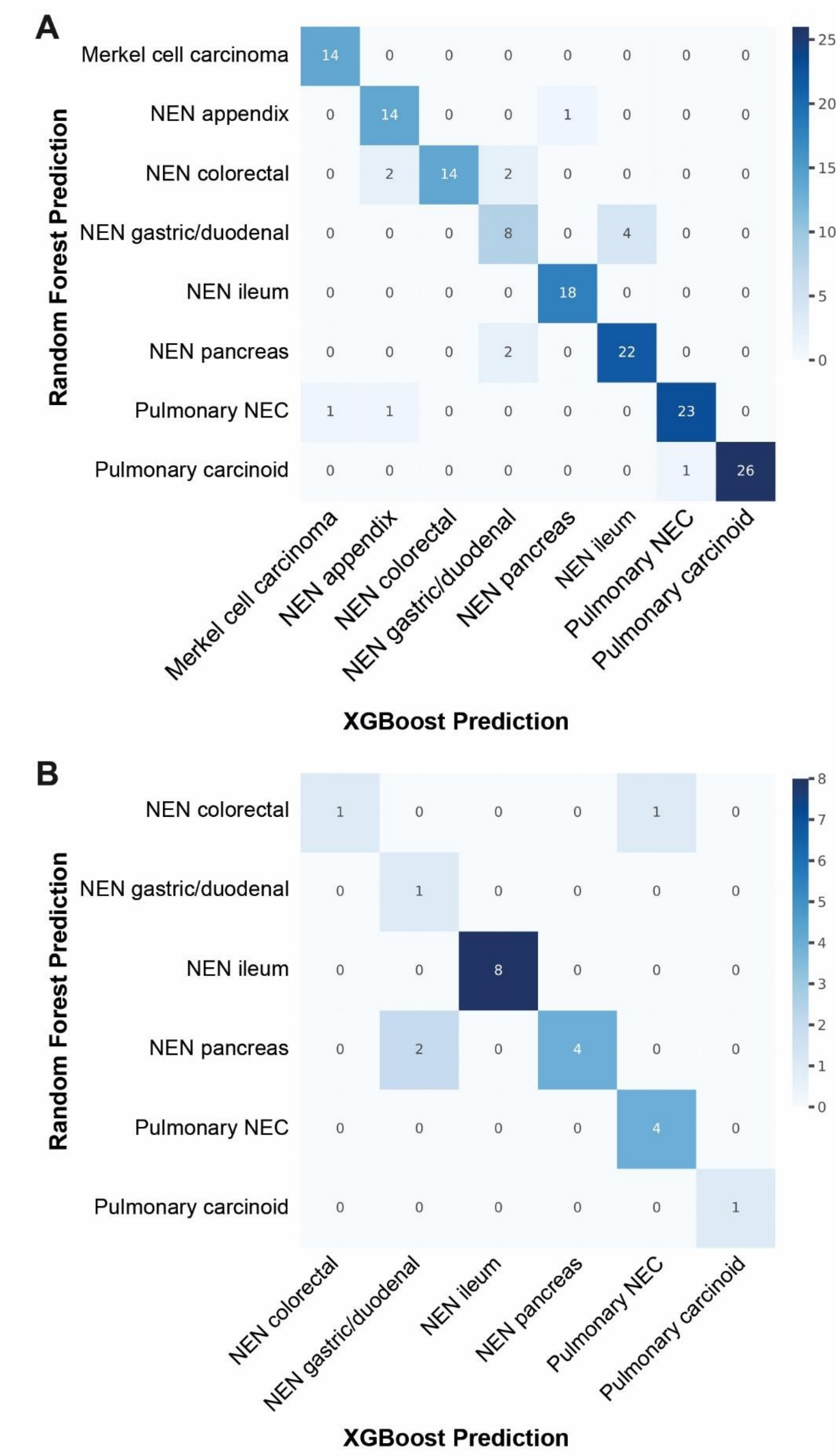

Supplementary Figure 11: Comparison of the Random Forest and XGBoost prediction algorithms.

(A) Confusion matrix showing the Random Forest prediction and XGBoost prediction of NEN the training set (N=153 samples) and **(B)** of the NEN liver metastases (N=22).

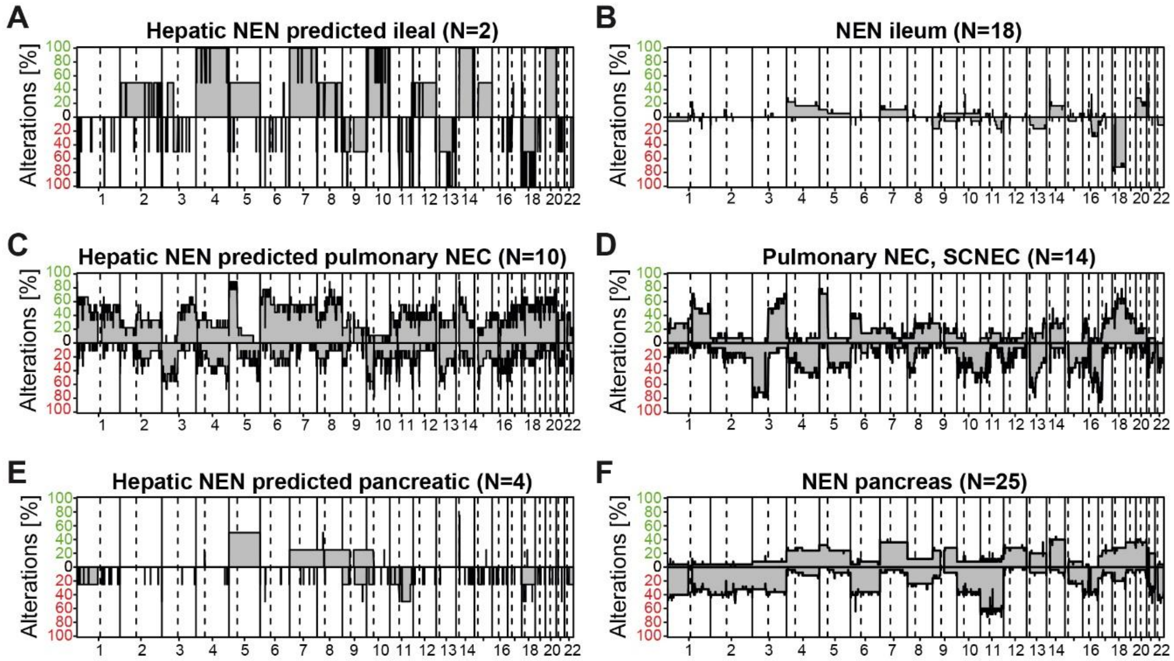

Supplementary Figure 12: Copy number profiles of primary NEN based on predicted organ site.

(A) Copy number alterations of hepatic NEN predicted to be derived from ileal NEN (N=2) and **(B)** of ileal NEN of the reference cohort (N=18). **(C)** Hepatic NEN predicted to be pulmonary NEC (N=10) and **(D)** primary pulmonary NEC (N=14) are depicted. **(E)** Copy number profiles of hepatic NEN predicted to be derived from pancreatic NEN (N=4) and **(F)** of primary pancreatic NEN of the reference cohort (N=25). The relative frequency of observed gains (green) and losses (red) are depicted above and below the horizontal line, respectively. SCNEC: small-cell neuroendocrine carcinoma.

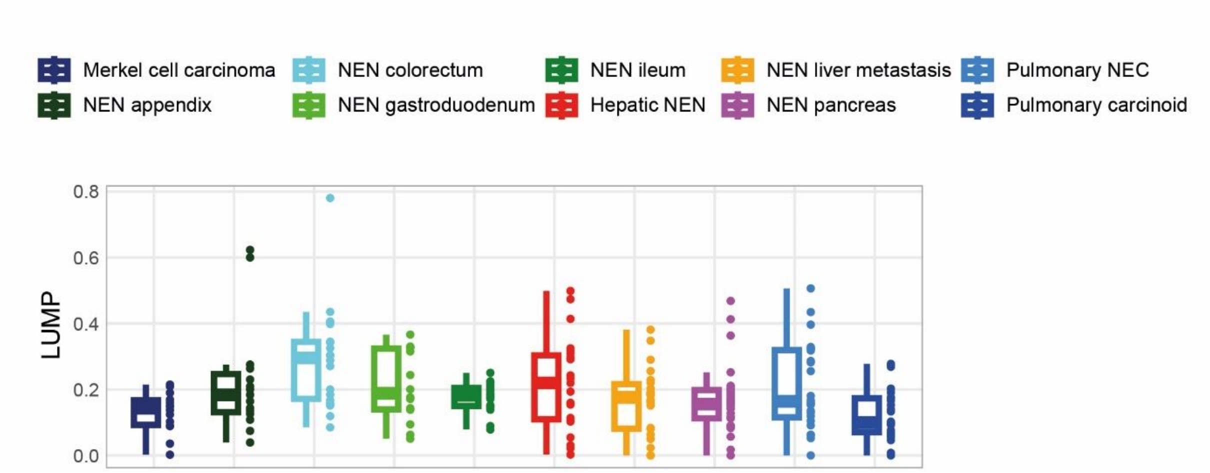

Supplementary Figure 13: Proportion of Leukocytes Unmethylation for Purity (LUMP) in NEN of different organ sites.

LUMP values in the DNA methylation profiles of Merkel cell carcinoma, colorectal NEN, Merkel cell carcinomas (N=14), appendiceal NEN (N=15), colorectal NEN (N=18), gastric/duodenal (N=14), ileal NEN (N=18), hepatic NEN without known primary tumor (N=27), NEN liver metastases of known primary (N=18), pancreatic NEN (N=25), pulmonary NEC (N=24) and pulmonary carcinoids (N=25), as indicated.
