# Supplementary material for: DNA methylation patterns facilitate tracing the origin of neuroendocrine neoplasms
Source: Nat Commun. 2025 Oct 27;16:9477. doi: 10.1038/s41467-025-65227-8 (PMC12559432; doi:10.1038/s41467-025-65227-8)
Supplement: Supplementary file 2 — Descriptions of Addtional Supplementary Files [file 41467_2025_65227_MOESM2_ESM.pdf]

**Description of Additional Supplementary Files**

**Supplementary Data 1.** Detailed immunohistochemical analyses of NEN groups (N=50)

**Supplementary Data 2.** Immunohistochemical analyses of NET G3 and NEC (N=32)

**Supplementary Data 3.** Detailed immunohistochemical analyses of hepatic NEN without known primary (N=22)

**Supplementary Data 4.** Immunohistochemical characterization of independent hepatic NEN validation cohort from Paris (N=14)

**Supplementary Data 5.** Overview of tissue samples including Leukocytes Unmethylation for Purity (LUMP)

**Supplementary Data 6.** Predicted origin of liver metastases of extrahepatic NEN (N=22)

**Supplementary Data 7.** Predicted origin of hepatic NEN of the validation cohort (N=15)

**Supplementary Data 8.** Antibodies used for immunohistochemical staining
