# Supplementary material for: DNA methylation patterns facilitate tracing the origin of neuroendocrine neoplasms
Source: Nat Commun. 2025 Oct 27;16:9477. doi: 10.1038/s41467-025-65227-8 (PMC12559432; doi:10.1038/s41467-025-65227-8)
Supplement: Supplementary file 11 — Reporting Summary [file 41467_2025_65227_MOESM11_ESM.pdf]

nature portfolio

Corresponding author(s): Stephanie Roessler
Benjamin Goeppert

Last updated by author(s): Sep 25, 2025

Reporting Summary

Nature Portfolio wishes to improve the reproducibility of the work that we publish. This form provides structure for consistency and transparency in reporting. For further information on Nature Portfolio policies, see our Editorial Policies and the Editorial Policy Checklist.

Statistics

For all statistical analyses, confirm that the following items are present in the figure legend, table legend, main text, or Methods section.

n/a	Confirmed	
☐	☒	The exact sample size (*n*) for each experimental group/condition, given as a discrete number and unit of measurement
☐	☒	A statement on whether measurements were taken from distinct samples or whether the same sample was measured repeatedly
☐	☒	The statistical test(s) used AND whether they are one- or two-sided<br>*Only common tests should be described solely by name; describe more complex techniques in the Methods section.*
☐	☒	A description of all covariates tested
☒	☐	A description of any assumptions or corrections, such as tests of normality and adjustment for multiple comparisons
☐	☒	A full description of the statistical parameters including central tendency (e.g. means) or other basic estimates (e.g. regression coefficient) AND variation (e.g. standard deviation) or associated estimates of uncertainty (e.g. confidence intervals)
☐	☒	For null hypothesis testing, the test statistic (e.g. *F*, *t*, *r*) with confidence intervals, effect sizes, degrees of freedom and *P* value noted<br>*Give P values as exact values whenever suitable.*
☒	☐	For Bayesian analysis, information on the choice of priors and Markov chain Monte Carlo settings
☒	☐	For hierarchical and complex designs, identification of the appropriate level for tests and full reporting of outcomes
☐	☒	Estimates of effect sizes (e.g. Cohen's *d*, Pearson's *r*), indicating how they were calculated

Our web collection on statistics for biologists contains articles on many of the points above.

Software and code

Policy information about availability of computer code

Data collection	n/a
Data analysis	R version 4.1, RnBeads version 2.10.0 including Greedycut, minfi package, methylumi package, MeDeCom, limma, scikit-learn Python version 3.8, code is available at https://github.com/Multi-omics-Data-Science-LIH/NEN-methylation

For manuscripts utilizing custom algorithms or software that are central to the research but not yet described in published literature, software must be made available to editors and reviewers. We strongly encourage code deposition in a community repository (e.g. GitHub). See the Nature Portfolio guidelines for submitting code & software for further information.

Data

Policy information about availability of data

All manuscripts must include a data availability statement. This statement should provide the following information, where applicable:

- Accession codes, unique identifiers, or web links for publicly available datasets
- A description of any restrictions on data availability
- For clinical datasets or third party data, please ensure that the statement adheres to our policy

All relevant data is included in the manuscript, supplementary material or made publicly available. The Infinium MethylationEPIC BeadChip data generated in this study are available at the Gene Expression Omnibus (GEO) repository under accession GSE253176 and GSE298677.

Research involving human participants, their data, or biological material

Policy information about studies with human participants or human data. See also policy information about sex, gender (identity/presentation), and sexual orientation and race, ethnicity and racism.

Reporting on sex and gender	Sex and gender were not analyzed in this study as male and female patients were similarly affected.
Reporting on race, ethnicity, or other socially relevant groupings	Race, ethnicity, or other socially relevant groupings were not reported.
Population characteristics	See above
Recruitment	N/A
Ethics oversight	The study protocol was approved by the ethics committee of Heidelberg University.

Note that full information on the approval of the study protocol must also be provided in the manuscript.

Field-specific reporting

Please select the one below that is the best fit for your research. If you are not sure, read the appropriate sections before making your selection.

☒ Life sciences ☐ Behavioural & social sciences ☐ Ecological, evolutionary & environmental sciences

For a reference copy of the document with all sections, see nature.com/documents/nr-reporting-summary-flat.pdf

Life sciences study design

All studies must disclose on these points even when the disclosure is negative.

Sample size	No sample size calculation was performed. As hepatic NEN without known primary are rare all samples available were included.
Data exclusions	No data was excluded.
Replication	Data analysis was independently performed by Reka Toth, Yue Zhang and Danial Tabbakh. All findings were replicated.
Randomization	Stratified 3-fold cross-validation was performed to evaluate the classifier's accuracy. Receiver Operating Characteristic (ROC) curves were plotted for each class using stratified 3-fold cross-validation and the area under the ROC curve (AUC) was computed.
Blinding	Blinding was not applied to the reference set as these groups were used for classifier building and hepatic NEN were unblinded after initial blinding as a result of the finding of heterogeneity.

Reporting for specific materials, systems and methods

We require information from authors about some types of materials, experimental systems and methods used in many studies. Here, indicate whether each material, system or method listed is relevant to your study. If you are not sure if a list item applies to your research, read the appropriate section before selecting a response.

Materials & experimental systems

n/a	Involved in the study	
☐	☒	Antibodies
☒	☐	Eukaryotic cell lines
☒	☐	Palaeontology and archaeology
☒	☐	Animals and other organisms
☐	☒	Clinical data
☒	☐	Dual use research of concern
☒	☐	Plants

Methods

n/a	Involved in the study	
☒	☐	ChIP-seq
☒	☐	Flow cytometry
☒	☐	MRI-based neuroimaging

Antibodies

Antibodies used	All antibodies used and the specific details including dilution are provided in Supplementary Data 8

Validation	All antibodies used in this study were validated for routine pathological diagnostics.

Clinical data

Policy information about clinical studies

All manuscripts should comply with the ICMJE guidelines for publication of clinical research and a completed CONSORT checklist must be included with all submissions.

Clinical trial registration	n/a
Study protocol	n/a
Data collection	n/a
Outcomes	n/a

Plants

Seed stocks	n/a
Novel plant genotypes	n/a
Authentication	n/a
